# Dynamin inhibitors induce caspase-mediated apoptosis following cytokinesis failure in human cancer cells and this is blocked by Bcl-2 overexpression

**DOI:** 10.1186/1476-4598-10-78

**Published:** 2011-06-28

**Authors:** Sanket Joshi, Antony W Braithwaite, Phillip J Robinson, Megan Chircop

**Affiliations:** 1 Children's Medical Research Institute, The University of Sydney, 214 Hawkesbury Road, Westmead, NSW 2145, Australia; 2 Department of Pathology, Dunedin School of Medicine, University of Otago, 364 Leith Walk, Dunedin 9016, New Zealand; 3 Tumour Cell Death Laboratory, Beatson Institute for Cancer Research, Garscube Estate, Switchback Rd, Glasgow, G61 1BD, UK

## Abstract

**Background:**

The aim of both classical (e.g. taxol) and targeted anti-mitotic agents (e.g. Aurora kinase inhibitors) is to disrupt the mitotic spindle. Such compounds are currently used in the clinic and/or are being tested in clinical trials for cancer treatment. We recently reported a new class of targeted anti-mitotic compounds that do not disrupt the mitotic spindle, but exclusively block completion of cytokinesis. This new class includes MiTMAB and OcTMAB (MiTMABs), which are potent inhibitors of the endocytic protein, dynamin. Like other anti-mitotics, MiTMABs are highly cytotoxic and possess anti-proliferative properties, which appear to be selective for cancer cells. The cellular response following cytokinesis failure and the mechanistic pathway involved is unknown.

**Results:**

We show that MiTMABs induce cell death specifically following cytokinesis failure via the intrinsic apoptotic pathway. This involves cleavage of caspase-8, -9, -3 and PARP, DNA fragmentation and membrane blebbing. Apoptosis was blocked by the pan-caspase inhibitor, ZVAD, and in HeLa cells stably expressing the anti-apoptotic protein, Bcl-2. This resulted in an accumulation of polyploid cells. Caspases were not cleaved in MiTMAB-treated cells that did not enter mitosis. This is consistent with the model that apoptosis induced by MiTMABs occurs exclusively following cytokinesis failure. Cytokinesis failure induced by cytochalasin B also resulted in apoptosis, suggesting that disruption of this process is generally toxic to cells.

**Conclusion:**

Collectively, these data indicate that MiTMAB-induced apoptosis is dependent on both polyploidization and specific intracellular signalling components. This suggests that dynamin and potentially other cytokinesis factors are novel targets for development of cancer therapeutics.

## Background

Drugs that disrupt mitotic progression are commonly referred to as 'anti-mitotics' and are extensively used for the treatment of cancer. Classical 'anti-mitotic' chemotherapeutics used in the clinic target microtubules and include the taxanes and vinca alkaloids [1]. Despite success in the clinic, drug resistance and toxicity have limited their effectiveness, due to the broad role of tubulin in the cytoskeleton of normal and non-dividing cells [1]. A new class of anti-mitotics have been developed that specifically target mitotic proteins such as Aurora kinase, polo-like kinase, kinesin spindle protein [1,2]. Such inhibitors are being characterised as potential chemotherapeutic agents since several induce mitotic failure leading to apoptotic cell death in cancer cells and xenograft mouse cancer models [2,3]. These mitotic proteins are either expressed only in dividing cells or apparently function exclusively during mitosis. In contrast to classical anti-mitotics, non-dividing differentiated cells should not be affected by such targeted inhibition, and thus they are predicted to be more efficacious. Many of these targeted inhibitors are currently in cancer clinical trials. Despite the differences in the protein being targeted, both classical and targeted anti-mitotics developed to date aim to disrupt the mitotic spindle or an early stage in mitosis.

We have recently reported a new class of targeted anti-mitotics that do not perturb the mitotic spindle but exclusively block cytokinesis [4]. The targeted protein for inhibition is the endocytic protein, dynamin II (dynII). DynII is best known for its role in membrane trafficking processes, specifically in clathrin-mediated endocytosis [5-7]. However, dynII also plays an essential role in the completion of the final stage of mitosis, cytokinesis [4-6,8-12]. We and others have developed several classes of dynamin inhibitors including dynasore [13], dimeric tyrphostins (Bis-Ts), long chain amines and ammonium salts (MiTMABs (myristyl trimethyl ammonium bromides)), dynoles [14-16], iminodyns [17] and pthaladyns [18]. Characterisation of the two most potent MiTMABs, MiTMAB and OcTMAB (collectively referred to as MiTMABs), revealed that they block the abscission phase of cytokinesis causing polyploidization, which is analogous to the dynII siRNA phenotype [4,8]. The MiTMAB dynamin inhibitors share many favourable characteristics with inhibitors of Aurora kinases, Plk and KSP: (i) they do not affect any other phase of the cell division cycle and (ii) possess anti-proliferative and cytotoxic properties that are selective for cancer cells [4]. Thus, targeting cytokinesis with dynamin inhibitors may be a promising new approach for the treatment of cancer.

Apoptotic cell death is central to targeted anti-mitotic compounds being highly efficacious as chemotherapeutic agents and is thought to depend on their ability to cause mitotic failure and subsequent accumulation of polyploid cells [3,19-21]. The mechanism of apoptosis following mitosis failure is poorly understood. It is thought to be classical apoptosis, involving caspase activation and poly(ADP-ribose) polymerase 1 (PARP1) cleavage [22]. However, cell death induced by caspase-independent mechanisms has been reported [23,24]. Apoptotic cell death does not always result following mitotic failure induced by an anti-mitotic. Various cellular responses, depending on the cell line and inhibitor analysed have been reported and include apoptosis, senescence and reversible mitotic arrest [25]. An in-depth understanding of the mechanisms driving a particular cellular fate in response to targeted anti-mitotics is crucial for rational development and their potential application as chemotherapeutic agents.

In this study, we aimed to determine the fate of cells and the signalling mechanisms involved following treatment with MiTMABs, which exclusively block abscission during cytokinesis. We report that MiTMABs induce cell death following cytokinesis failure in several cancer cells and this was mediated by the intrinsic apoptotic pathway. The cellular response of cancer cells to MiTMABs appeared to correlate with expression of Bcl-2. Our results indicate that the anti-proliferative and cytotoxic properties of the MiTMAB dynamin inhibitors are due to their ability to induce apoptosis following cytokinesis failure. This provides the first evidence that targeting cytokinesis is a valid approach for the development of anti-cancer agents, and that dynII inhibitors are the first class of compounds in this new targeted anti-mitotic group.

## Methods

### Cell culture

HeLa, HeLa-Bcl-2 [26] and H460 cell lines were maintained in RPMI 1640 medium supplemented with 10% foetal bovine serum (FBS) and 5% (P/S). HT29, SW480 and MCF-7 cell lines were maintained in Dulbecco's Modified Eagle's Medium (DMEM) supplemented with 10% FBS and 5% P/S. All cells were grown at 37 °C in a humidified 5% CO_2 _atmosphere.

### Drugs

The active dynamin inhibitors, MiTMAB (also known as tetradecyl trimethyl ammonium bromide, CAS number 119-97-7), OcTMAB (CAS number 1120-02-1; Sigma-Aldrich, Co., St. Louis, MO), and the inactive analogue, 2-(DiMA)EM (2-(dimethylamino) ethyl myristate; Lancaster Synthesis, England), were prepared as 30 mM stock solutions in DMSO and stored at -20°C. Cytochalasin B (cytB) was prepared as 5 mg/ml stock solutions in DMSO and stored at -20°C. The CDK1 small molecule inhibitor RO-3306 was synthesised in-house as reported previously [4]. Stock solution (9 mM) of RO-3306 was prepared in DMSO and stored at -20°C. The pan-caspase inhibitor Z-VAD-FMK (ZVAD) and the caspase-8 selective inhibitor Z-IETD-FMK (IETD) were purchased from BD Biosciences and used at a final concentration of 50 µM.

### Cell synchronization and treatment with MiTMABs

Cells were synchronized at the G_2_/M boundary by treatment with RO-3306 (9 µM) for 18 hours [4,8] and at the G_1_/S boundary by the double thymidine block assay [27] as previously described. Immediately following RO-3306 or thymidine removal, cells synchronously entered the cell cycle and were treated with MiTMABs. As a negative control, cells were released into drug-free medium, or medium containing 0.1% DMSO or the inactive analogue 2-(DiMA)EM. As a positive control for apoptosis, cells were irradiated with ultraviolet (UV-C) light at 100 J/m^2^.

### Cell cycle analysis by flow cytometry

Cells (5 × 10^5 ^cells per dish) were grown in 10 cm dishes. Following inhibitor treatment, cells (floating and adherent) were collected and single-cell suspensions were fixed in 80% ice-cold ethanol at -20 °C for at least 16 hours. Cells were stained with propidium iodide and cell cycle was analysed [4]. Cell cycle profiles were acquired with a FACS Canto Flow Cytometer (Becton Dickinson) using FACS Diva software (v.5.0.1) at 488 nm. Cell cycle profiles were analysed using FlowJo software (v.7.1).

Where indicated, the drugs were removed by washing three times with drug-free medium after a 6 h treatment. Cells were then incubated for an additional 42 h in drug-free medium prior to fixation and flow cytometry analysis.

### Time-lapse analysis

Cells were seeded in 6-well plates (1 × 10^5 ^cells per well) and synchronized at the G_2_/M boundary as described above. Immediately following release into the cell cycle, cells were treated with the indicated molecule and viewed with an Olympus IX80 inverted microscope. A time-lapse series was acquired using a fully motorised stage, 10x objective, and Metamorph software using the time-lapse modules. Temperature was controlled at 37 °C using the Incubator XL, providing a humidified atmosphere with 5% CO_2_. Images were captured every 10 minutes for 20 hours. Where indicated, a time-lapse series was acquired in asynchronously growing cells immediately following the addition of the indicated drug.

### Immunofluorescence microscopy

Cells were fixed in ice-cold 100% methanol and immunostaining was carried using the anti-α-tubulin (Clone DM1A; Sigma) antibody [4,27]. Cells were viewed and scored for multinucleation with a fluorescence microscope (Olympus BX51). Fluorescence images were captured and processed using an Olympus IX80 inverted microscope using 40x or 100x oil immersion lenses and Metamorph software. Images were deconvolved using AutoDeblur v.9.3 (AutoQuant Imaging, Watervliet, NY).

### Immunoblotting

Cell lysates were prepared as described previously [28]. In brief, cells were collected by centrifugation, washed with PBS, then resuspended in ice-cold lysis buffer (20 mM Tris-HCl (pH 7.4), 150 mM NaCl, 1 mM EDTA, 1 mM EGTA, 1% Triton X-100 and EDTA-free Complete protease inhibitor cocktail (Roche)) for 30 mins. The supernatant (cell lysate) was collected following centrifugation at 13,000 rpm for 30 min at 4ºC. Cell lysates (50 μg) were fractionated by SDS-PAGE for immunoblot analysis using the following primary antibodies: Bcl-2, Bcl-XL, Mcl-1, cleaved caspase-8, -9, -3, PARP (Cell Signaling Tech) and β-actin (Sigma-Aldrich). Primary antibody was detected by incubation with horseradish peroxidise-conjugated anti-rabbit or anti-mouse secondary antibody (Jackson ImmunoResearch Laboratories). Blotted proteins were visualized using the ECL chemiluminescence detection system (Pierce).

## Results

### HeLa cells undergo apoptosis following cytokinesis failure

**Figure 1:**
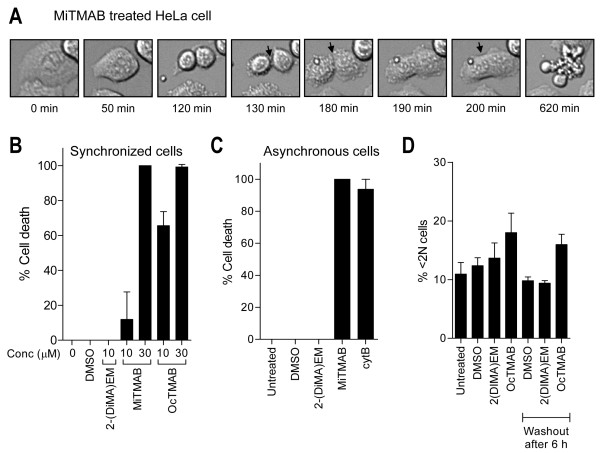
**MiTMABs induce apoptosis following cytokinesis failure in Hela cells**. A-B, HeLa cells were released from the G_2_/M boundary in the presence of MiTMAB, OcTMAB or controls (untreated, 0.1% DMSO and 2-(DiMA)EM) at the indicated concentrations. Cells were immediately monitored by time-lapse microscopy for 20 hours. A, Representative time-lapse images of a HeLa cell treated with 10 µM MiTMAB undergoing mitosis are shown. This cell undergoes apoptotic cell death, characterised by membrane blebbing, 420 min after failing cytokinesis. Cytokinesis failure was due to an inability to abscise the intracellular bridge, thus the cleavage furrow regressed resulting in formation of a binucleated cell. The percentage of binucleated HeLa cells that underwent apoptosis following cytokinesis failure are shown (B). Graph represents mean ± S.D. where n > 160 for each experimental condition. C, Asynchronously growing HeLa cells were monitored by time-lapse microscope for 20 h following addition of 30 µM MiTMAB, 4.5 µg/ml cytochalsin B (cytB) or controls (untreated, 0.1% DMSO and 2-(DiMA)EM). The graph represents mean ± S.D. of the percentage of cytokinesis failed HeLa cells that underwent apoptosis. D, G_2_/M synchronized HeLa cells were analysed after 48 h following exposure to OcTMAB and controls (untreated, 0.1% DMSO and 2-(DiMA)EM) for the entire duration or for only the first 6 h (removal by washout). The graph (mean ± S.D. from two independent experiments) shows the percentage of apoptotic cells as indicated by <2N DNA content using flow cytometry.

MiTMABs inhibit cell proliferation and reduce viability in a range of cancer cells [4]. In HeLa cells these effects were due to the ability of the MiTMABs to induce apoptosis. MiTMABs also cause polyploidization by inducing cytokinesis failure at the abscission stage [4]. Since induction of apoptosis by anti-mitotic compounds is thought to depend on polyploidization [3,19,20], we used time-lapse microscopy and individual cell analysis to ask if apoptosis follows multinucleation induced by MiTMABs. G_2_/M synchronized HeLa cells treated with MiTMABs progress through mitosis normally, enter cytokinesis and complete membrane ingression, as previously observed [4]. However, they fail at the abscission stage of cytokinesis resulting in cleavage furrow regression and formation of a binucleated cell (Figure 1A). Apoptotic cell death was observed approximately 420 mins following mitosis failure as indicated by membrane blebbing and formation of apoptotic bodies (Figure 1A). Among the cells treated with MiTMABs that failed cytokinesis, apoptosis occurred in a dose-dependent manner, with 100% of cells undergoing cell death at 30 µM (Figure 1B). In contrast, the inactive MiTMAB analogue, 2-(DiMA)EM, did not have a significant effect on cell death (Figure 1B). Similar results were obtained in asynchronous cells indicating no effect of the synchronization agent (Figure 1C). The results demonstrate that MiTMAB-induced apoptosis occurs primarily following cytokinesis failure. Cell death also occurred to a similar extent as MiTMAB treatment in those cells that had failed cytokinesis in the presence of the cytokinesis inhibitor, cytochalasin B (cytB; Figure 1C). Thus, failure of cytokinesis appears to be toxic to cells.

We next sought to determine when after cytokinesis failure the cells were committed to apoptosis by using flow cytometry. By 6 h after release from the G_2_/M boundary, the majority of cells have entered mitosis and completed this process albeit either successfully (two mononucleated daughter cells) or unsuccessfully (binucleated cell). At this time point, no morphological signs of apoptosis are evident. As expected, after a 48 h treatment period, OcTMAB induced apoptosis in G_2_/M synchronized cells, as evident by an increase in the percentage of cells with <2N DNA content (Figure 1D). Apoptosis was still evident in cells after 48 h when OcTMAB was removed by wash-out after only a short 6 h treatment (Figure 1D), indicating that the cells were already committed to cell death very soon after cytokinesis failure and binucleate formation. This again suggests that the induction of apoptosis is associated with cytokinesis failure and not due to generalised toxicity of the MiTMABs.

### HeLa cells undergo caspase-mediated apoptosis exclusively following cytokinesis failure

**Figure 2:**
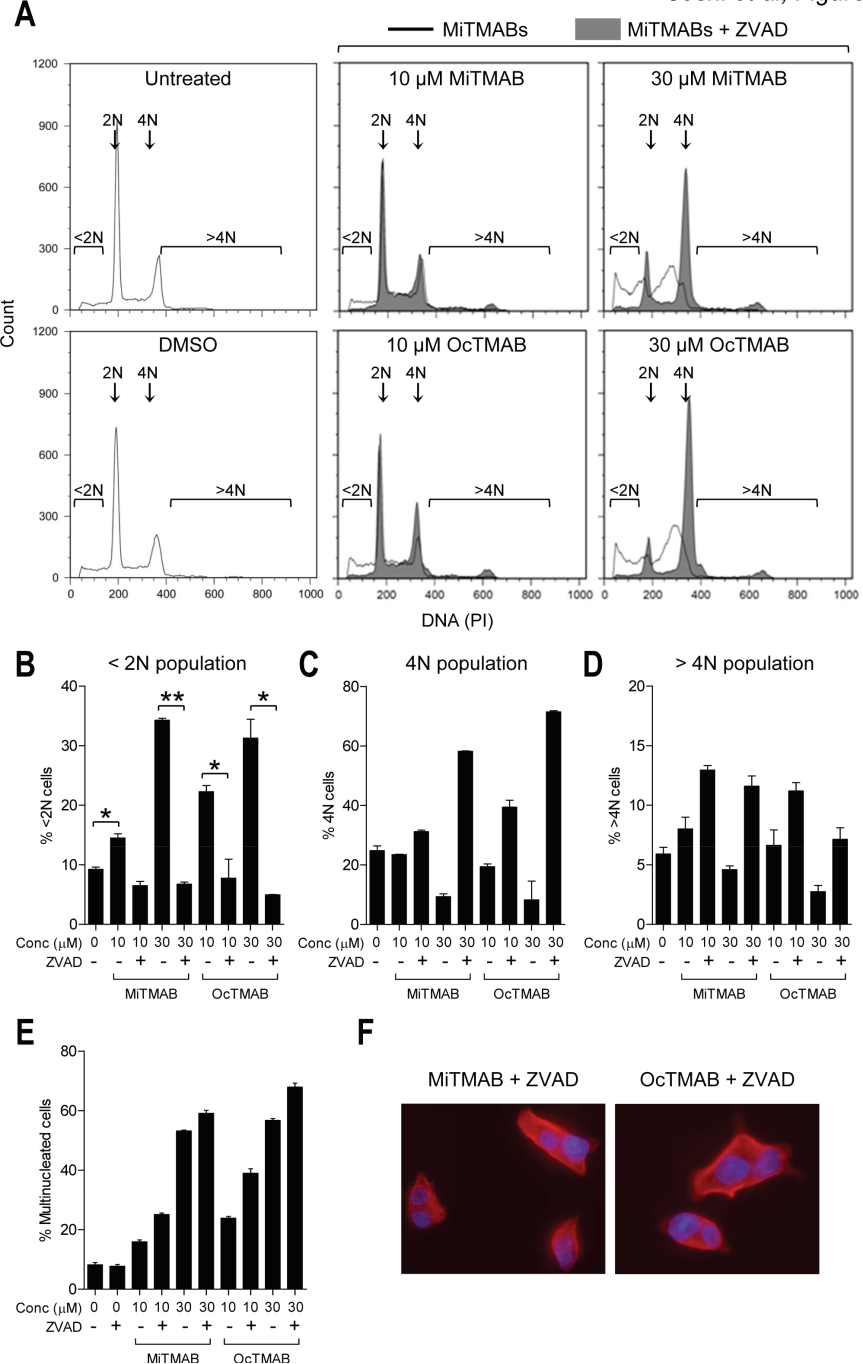
**Cell death induced by MiTMABs is blocked by the pan-caspase inhibitor, ZVAD**. A-D, G_2_/M synchronized HeLa cells were treated for 20 h with MiTMABs and controls (untreated, 0.1% DMSO and 2-(DiMA)EM) in the presence and absence of the pan-caspase inhibitor, ZVAD. DNA content in these cells was analysed by flow cytometry. Representative flow cytometry histograms of propidium iodide stained control treated (untreated and 0.1% DMSO) HeLa cells and HeLa cells treated with MiTMABs in the presence (filled histograms) and absence (empty histograms) of ZVAD are shown (A). The percentage of cells (mean ± S.D. from two independent experiments) with < 2N (DNA fragmentation, which is indicative of cells undergoing apoptosis; (B), 4N (C), and >4N (D) DNA content are shown. Treatment with MiTMABs + ZVAD caused a decline in cells containing <2N DNA and a corresponding increase in cells containing ≥ 4N DNA content compared to MiTMABs alone. * p < 0.05, ** p < 0.01 (Student's *t *tests). E, HeLa cells were treated as described in A except that after 8 h cells were fixed and stained for α-tubulin, and the percentage of cells that were multinucleated was scored using immunofluorescence microscopy. Graph shows mean ± S.D. from two independent experiments. F, Representative microscopy images of (E) illustrating multinucleated HeLa cells treated with either MiTMAB or OcTMAB in combination with ZVAD. Red, α-tubulin. DNA, blue.

Apoptosis is characterized by activation of a caspase-dependent pathway. Therefore, we aimed to confirm the activation of this pathway in response to MiTMABs and to characterize the molecular components. To confirm the caspase dependence we co-incubated MiTMABs with the pan-caspase inhibitor ZVAD and quantified apoptosis by flow cytometry. Treatment with ZVAD completely blocked apoptosis induced by 10 and 30 µM MiTMABs in G_2_/M synchronized HeLa cells (Figure 2A and 2B). Thus, the presence of ZVAD protects cells treated with MiTMABs from apoptosis. Consistent with apoptosis occurring post-cytokinesis failure, we observed a corresponding increase in the percentage of cells containing 4N and >4N DNA content in samples treated with MiTMABs and ZVAD compared to MiTMABs alone (Figure 2A, C and 2D). These cell populations increased with increasing concentrations of both MiTMABs (Figure 2C and 2D). Specifically, 6.6 ± 0.9% and 2.7 ± 0.4% of 10 and 30 µM OcTMAB-treated cells, respectively, contained >4N DNA and in the presence of ZVAD this increased to 11.2 ± 0.5% and 7.1 ± 0.7% of OcTMAB-treated cells, respectively. Immunofluorescence microscopy analysis confirmed that the cells containing ≥4N DNA were multinucleated and not trapped in G_2 _or mitosis phase of the cell cycle (Figure 2E and 2F). Consistent with the flow cytometry data, multinucleation increased in cells treated with both MiTMABs in a dose-dependent manner and was further increased in the presence of ZVAD (Figure 2E). This suggests that MiTMABs induce apoptosis via a caspase-dependent pathway and that apoptosis induced by MiTMABs occurs following cytokinesis failure.

**Figure 3:**
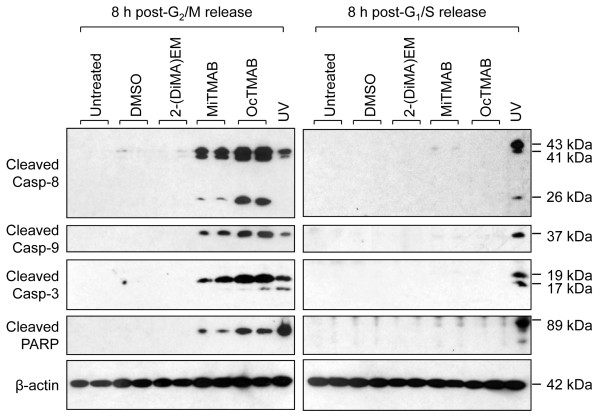
**MiTMABs induce caspase activation specifically in cells undergoing mitosis**. G_2_/M and G_1_/S synchronized cells were treated for 8 h with 10 µM MiTMAB, 10 µM OcTMAB or indicated controls (untreated, 0.1% DMSO and 10 µM 2-(DiMA)EM). The lysates (prepared in duplicate) were immunoblotted for cleaved caspase-8, -9, -3 and PARP. These cleaved products were observed in cells treated with MiTMABs that had been synchronized at the G_2_/M, but not the G_1_/S boundary. Lysates prepared from cells exposed to UV served as a positive control. β-actin levels were used as a loading control.

To identify the molecular pathway involved in executing apoptotic cell death mediated by MiTMABs following cytokinesis failure, we sought to detect activation of specific caspases. Time-lapse analysis revealed that G_2_/M synchronized cells enter mitosis within 1 h and complete this process within 2h following release from RO-3306 block (Figure 1A). In the presence of MiTMABs cells undergo mitosis with the same timing, but fail cytokinesis at approximately 3 h. Cell death indicated by membrane blebbing is observed approximately 7-8 h (10-11 h post-release from RO-3306 block) following cytokinesis failure (Figure 1A). Therefore, we harvested cells at 8 h post release from RO-3306 block (approx 5 h post-cytokinesis failure) to detect activation of caspases. Immunoblotting of MiTMABs-treated cell lysates revealed the presence of cleaved caspase-8, -9 and -3 and cleaved PARP (Figure 3), a target of caspase-3 in the molecular pathway driving apoptosis [29,30]. These proteins were also cleaved following exposure to UV as expected [31], but not after DMSO or 2-(DiMA)EM treatment, nor in untreated cells (Figure 3, left panels). In contrast to G_2_/M synchronized cells, caspase and PARP cleavage products were not detected in G_1_/S synchronized cells following exposure to identical MiTMAB treatment conditions (Figure 3, right panels). In this case, cells proceed through S phase but do not enter mitosis by 8 h and therefore cytokinesis failure does not occur. Thus, MiTMABs-induced caspase activation occurs exclusively following a mitotic division. In contrast, caspase and PARP cleavage was detectable in both synchronized cell populations exposed to UV (Figure 3). The results indicate that cell death induced by MiTMABs is a result of MiTMAB-induced cytokinesis failure and is mediated by a caspase-dependent pathway.

### HeLa cells stably expressing Bcl-2 are resistant to MiTMABs-induced cell death

**Figure 4:**
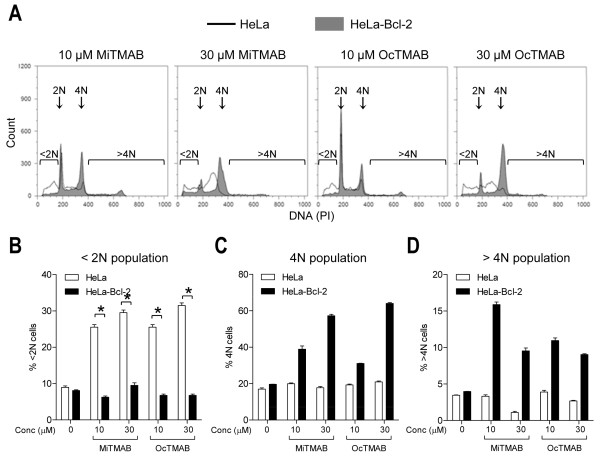
**Over-expression of Bcl-2 protects cells from MiTMABs-induced cell death**. A-D, G_2_/M synchronized HeLa (empty histograms) and HeLa-Bcl-2 (filled histograms) cells were synchronized at the G_2_/M boundary. Once released from this block, cells treated with MiTMAB, OcTMAB or indicated controls, were incubated for 20 h and their DNA contents analysed by flow cytometry. Representative flow cytometry histograms show a decrease in the <2N peak and an increase in the 4N peak in HeLa-Bcl-2 cells treated with MiTMAB (filled histograms) compared to parental HeLa cells (empty histograms; A). Graphs showing the percentage of HeLa and HeLa-Bcl-2 cells (mean ± S.D.) containing <2N (B), 4N (C) and >4N (D) DNA contents are shown. * p < 0.05, ** p < 0.01 (Student's *t *tests).

The activation of caspase-9 in MiTMABs-treated cells indicates that the intrinsic pathway is involved in mediating cell death. Caspase-9 is an initiator caspase activated following cytochrome c release from mitochondria [32]. Anti-apoptotic Bcl-2 family of proteins are directly responsible for maintaining mitochondrial membrane integrity, preventing cytochrome c release in the absence of apoptotic stimuli [33]. Therefore, we hypothesised that high Bcl-2 expression would inhibit MiTMAB-induced cell death. Indeed, flow cytometric quantitation of cells with <2N DNA content revealed that MiTMAB-induced apoptosis is completely blocked in HeLa cells stably expressing Bcl-2, HeLa-Bcl-2 (6.8 ± 0.3% in HeLa-Bcl-2 cells compared to 31.5 ± 0.5% in HeLa cells treated with 30 μM OcTMAB; Figure 4A and 4B). A corresponding increase in polyploid cells (4N and >4N DNA content) was observed (Figure 4C and 4D), further supporting the idea that cell death follows MiTMAB-induced cytokinesis failure. These results are analogous to those obtained in HeLa cells treated with the pan-caspase inhibitor, ZVAD (Figure 3A and 3B). We conclude that Bcl-2 over-expression renders HeLa cells resistant to MiTMAB-induced cell death, but not to MiTMAB-induced cytokinesis failure. The involvement of caspase-9 and Bcl-2 further indicate activation of the intrinsic apoptotic pathway.

### MiTMABs-induced cell death occurs via the intrinsic apoptotic pathway

**Figure 5:**
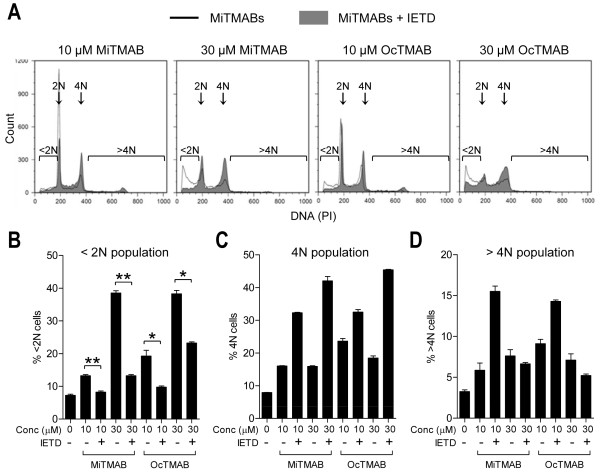
**The caspase-8 inhibitor, IETD, only partially blocks cell death induced by MiTMABs**. A-D, G_2_/M synchronized HeLa cells were treated with MiTMABs and controls as described in the legend to Figure 2, except that the caspase-8 inhibitor, IETD, was used instead of ZVAD. Representative flow cytometry histograms of HeLa cells treated with MiTMABs in the presence (filled histograms) and absence (empty histograms) of IETD are shown (A). Quantitation (mean ± S.D.) of cells containing <2N (B), 4N (C) and >4N (D) DNA contents were determined from two independent experiments. * p < 0.05, ** p < 0.01 (Student's *t *tests).

**Figure 6:**
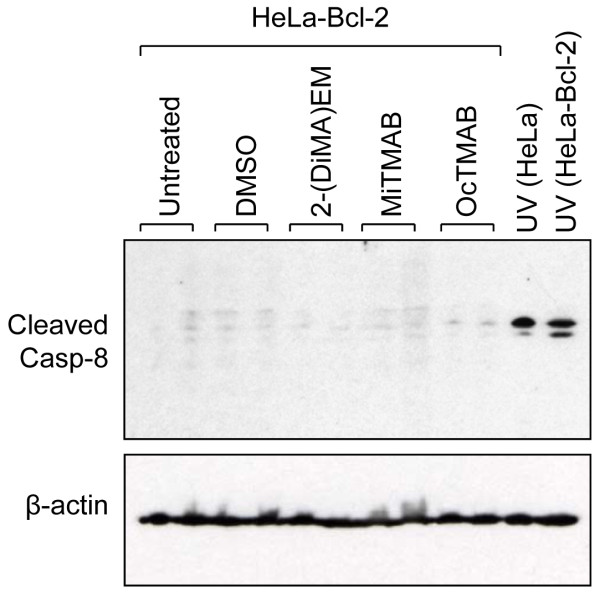
**Caspase-8 activation in MiTMAB treated cells occurs downstream of caspase-9/3 activation**. G_2_/M synchronized HeLa-Bcl-2 cells were treated with MiTMABs and controls as described in the legend to Figure 5. Following an 8 h exposure to MiTMABs, lysates (prepared in duplicate) were immunoblotted for caspase-8. In contrast to HeLa cells (Figure 3), cleaved caspase-8 was not observed in HeLa-Bcl-2 cells treated with MiTMABs. Lysates prepared from UV treated HeLa and HeLa-Bcl-2 cells were used as a positive control. β-actin served as a loading control.

The activation of another initiator caspase, caspase-8, was also detected in cells treated with MiTMABs. Unlike caspase-9, caspase-8 is a component of the extrinsic apoptotic pathway and is thus normally activated following stimulation of cell surface receptors [32]. Once activated, it cleaves the pro-apoptotic Bcl-2 family member, Bid, which in turn stimulates the intrinsic apoptotic pathway to promote cytochrome c release from mitochondria [34,35]. However, caspase-8 can also be activated by caspase-9/-3 in a feedback loop to amplify the already active intrinsic pathway [36]. Therefore, we sought to determine if activation of caspase-8 in response to MiTMABs occurs following stimulation of the extrinsic pathway (cell surface death receptors) and/or via intrinsic cell death signals. We first investigated the ability of MiTMABs to induce apoptosis in the presence of the caspase-8 selective inhibitor IETD. If the intrinsic pathway was solely induced by caspase-8, inhibiting caspase-8 alone should block cytochrome c release and subsequent cell death. However, inhibition of caspase-8 (IETD) only blocked apoptosis by approximately 40% (23.3 ± 0.3% HeLa cells treated with 30 μM OcTMAB + IETD compared to 38.3 ± 0.8% in HeLa cells treated with 30 μM OcTMAB alone; Figure 5A and 5B), in striking contrast to the effect of the pan-caspase inhibitor, ZVAD (Figure 2). IETD treatment also resulted in only a modest increase in polyploid cells (≥4N DNA content; Figure 5C and 5D), presumably because a significant proportion of cells that failed cytokinesis were able to undergo apoptosis. These findings suggest that activation of caspase-8 induced by MiTMABs is via the intrinsic pathway. Bcl-2 over-expression blocks cell death upstream of caspase-9 and -3 activation and thus caspase-8 cleavage should be prevented in HeLa-Bcl-2 cells if it is activated exclusively via the intrinsic pathway. In line with this idea, we did not detect cleaved caspase-8 in MiTMAB-treated HeLa-Bcl-2 cells (Figure 6). In contrast, caspase-8 cleavage was detected in both HeLa and HeLa-Bcl-2 cells exposed to UV, a known stimulant of the extrinsic pathway [31]. We conclude that MiTMABs induce apoptosis via the intrinsic apoptotic pathway and this involves activation of caspase-8 via a feedback amplification loop.

### The apoptotic response of cancer cells to MiTMABs appears to correlate with expression of Bcl-2 and Mcl-1 anti-apoptotic proteins

**Figure 7:**
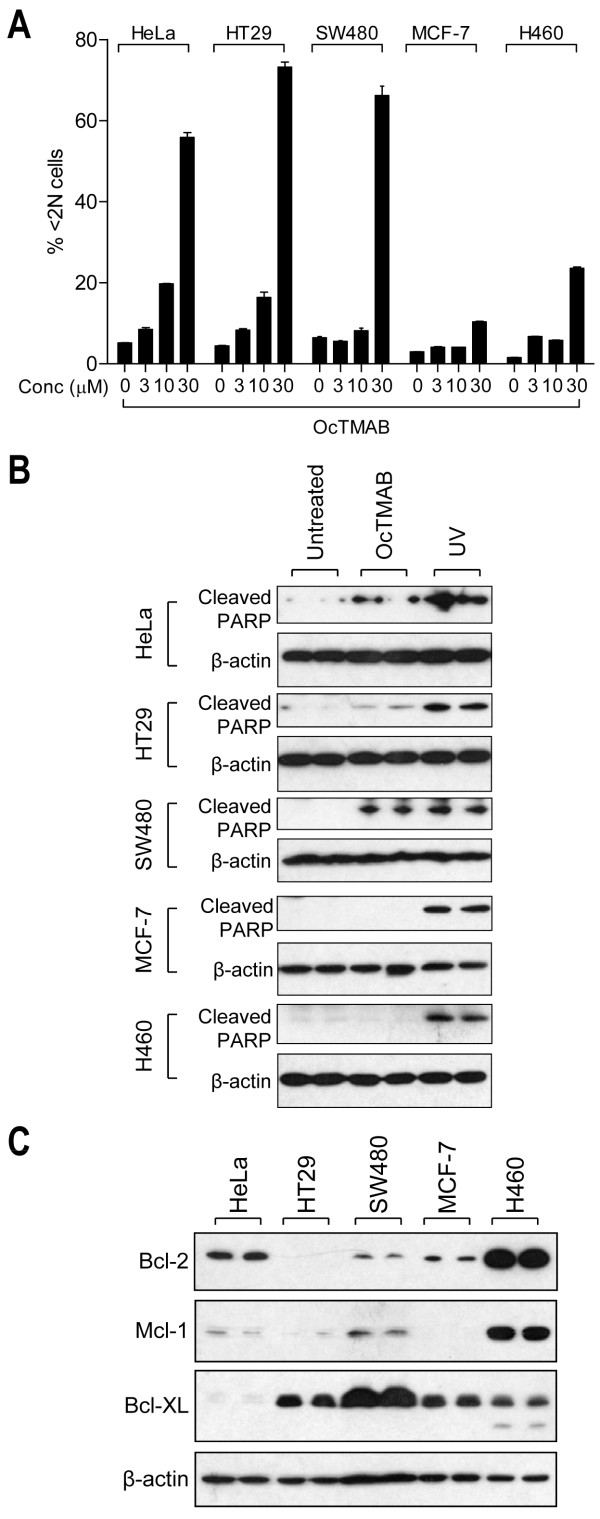
**Cancer cells expressing high levels of Bcl-2 are resistant to apoptosis induced by MiTMABs**. A-B, Asynchronously growing HeLa, HT29, SW480, MCF-7 and H460 cells were treated with the indicated concentration of OcTMAB for 48 h. Cells containing <2N DNA content (mean ± S.D. from two independent experiments) were determined by flow cytometry (A) and the presence of cleaved PARP was assessed by immunoblotting (B). C, Expression levels of the Bcl-2 family members, Bcl-2, Mcl-1 and Bcl-XL, were assessed and compared in the indicated cancer cell lines by immunoblotting. β-actin served as a loading control.

We next aimed to confirm if MiTMABs induce apoptosis in other cancer cell lines. We first analysed the cell cycle profile by flow cytometry following a 48 h treatment with OcTMAB of five cancer cell lines derived from different tissues: HeLa (cervical), HT29 and SW480 (colon), MCF-7 (breast) and H460 (lung). A significant increase in apoptosis (<2N DNA content) was observed in three of the cell lines (HeLa, HT29 and SW480) following exposure to OcTMAB (Figure 7A). Apoptosis increased in a dose-dependent manner with up to >70% of HT29 cells undergoing apoptosis when exposed to 30 µM OcTMAB (Figure 7A). In contrast, MCF-7 and H460 cells were largely resistant to OcTMAB-induced apoptosis with only 10.4 ± 0.1% and 23.6 ± 0.2% of cells, respectively, having <2N DNA content at 30 µM. PARP cleavage occurred in HeLa, HT29 and SW480 cells following exposure to OcTMAB but not in MCF-7 and H460 cells (Figure 7B), consistent with the flow cytometry data. In contrast, PARP cleavage occurred in all five cell lines following exposure to UV (Figure 7B). This is not surprising, as unlike MiTMABs, UV can trigger apoptosis via both the intrinsic and extrinsic pathways [31]. We conclude that MiTMABs induce apoptosis via a caspase-dependent mechanism in a range of cancer cells.

We next sought to gain insight into why specific cancer cells are sensitive (HeLa, SW480 and HT29) and others are resistant (H460 and MCF-7) to apoptosis induced by MiTMABs. We showed that HeLa cells stably expressing the anti-apoptotic protein, Bcl-2, are resistant to apoptosis induced by MiTMABs. Moreover, Bcl-2 family members are frequently over-expressed in cancers and confer resistance to anti-mitotic chemotherapy in various tumour types [37,38]. Therefore, we analysed the expression levels of three anti-apoptotic Bcl-2 family members, Bcl-2, Bcl-XL and Mcl-1, in all five cancer cell lines. Immunoblotting revealed that the three lines which are sensitive to MiTMABs, HeLa, HT29 and SW480, have relatively low levels of Bcl-2 and Mcl-1 (Figure 7C), which correlated well with the ability of MiTMABs to induce apoptosis in these cells. Although the MiTMABs-resistant MCF-7 cells also expressed low levels of these proteins (Figure 7C), their resistance can likely be explained by their underlying deficiency in caspase-3 [39]. In contrast, high levels of Bcl-2 and Mcl-1 proteins were detected in H460 cells (Figure 7C). Again, this correlated well with resistance of this cell line to MiTMABs-induced apoptosis. Except for HeLa cells, which expressed almost undetectable levels of Bcl-XL, the other four cell lines expressed moderate levels (Figure 7C). Thus, unlike Bcl-2 and Mcl-1, Bcl-XL protein levels did not correlate well with sensitivity to MiTMABs. The results suggest that the ability of MiTMABs to induce apoptosis appears to be dependent on the relative expression levels of the anti-apoptotic proteins Bcl-2 and Mcl-1.

## Discussion

Dynamin inhibitors are a new class of targeted anti-mitotic compounds. In contrast to the classical (e.g. taxol) and known targeted (e.g. Aurora kinase and Plk inhibitors) anti-mitotic compounds which aim to disrupt the mitotic spindle, the MiTMAB dynamin inhibitors exclusively block cytokinesis without disrupting progression through any other stage of mitosis. Analogous to other anti-mitotic compounds, dynamin inhibitors also have putative anti-tumour activity [4]. In this study, we show that two dynamin inhibitors called the MiTMABs induce cytokinesis failure and induce apoptosis in cancer cells and this appears to correlate with low expression of the anti-apoptotic proteins Bcl-2 and Mcl-1. Apoptosis occurred strictly following formation of a polyploid cell and was mediated via the intrinsic pathway. Over-expression of the anti-apoptotic protein, Bcl-2, blocked MiTMAB-induced apoptosis but not polyploidization. The induction of apoptosis exclusively following mitotic damage is analogous to the effect of targeted anti-mitotics, such as aurora kinase and Plk inhibitors [1]. We also demonstrate that apoptosis is induced in cells that have failed cytokinesis due to treatment with the cytokinesis blocker, cytochalsin B. Therefore, this is the first study to demonstrate that cytokinesis blockers can specifically induce apoptotic cell death and thus represent a new class of anti-mitotics with potential anti-cancer activity. Our results indicate that dynamin II is the primary target in this new anti-mitotic action.

Cells exposed to MiTMAB undergo cell death via activation of the intrinsic apoptotic pathway. This was evident by the presence of cleaved caspase-3, -9, and PARP, an increase in DNA fragmentation (<2N DNA content), and membrane blebbing. We further demonstrate that this intrinsic apoptotic pathway involves a feedback caspase-8 amplification loop to drive the execution of apoptosis. MiTMAB-induced cell death exclusively occurred following cytokinesis failure and subsequent polyploidization. This was demonstrated by several findings. Independent single cell analysis using time-lapse microscopy revealed that those MiTMAB-treated cells that failed cytokinesis subsequently underwent apoptotic cell death. We observed an increase in polyploidization in MiTMAB-treated cells when apoptosis was blocked by ZVAD or Bcl-2 overexpression. Caspase-8, -9, -3 and PARP cleavage products were not observed in cells treated with MiTMABs that were not able to undergo a mitotic division (8 h treatment from G_1_/S synchronization). Similar reports of cell death specifically following polyploidization in the presence of targeted inhibitors, such as aurora kinase, Plk and KSP inhibitors, have been reported [1,2,40]. This indicates that inhibition of a specific target is not the trigger for apoptosis but rather that it is the phenotype or subsequent molecular alteration generated as a result of its disruption.

The ability of anti-mitotic compounds to induce apoptosis exclusively in dividing cells is the primary rationale that they may be efficacious chemotherapeutic compounds [3,19,20,41]. However, an increased level of polyploidization does not appear to translate into increased level of secondary apoptosis [42]. Rather the resulting induction of apoptosis appears to be cell type specific. In line with this idea, the cellular response following exposure to a particular anti-mitotic varies and includes not only apoptosis, but also mitotic catastrophe, senescence and reversible mitotic arrest [25]. One determinant thought to predict the cellular response to a particular anti-mitotic is the time spent blocked in mitosis [43]. In the presence of the microtubule-stabilising drugs, ZM447439 (Aurora A/B inhibitor) and taxol, cells blocked in mitosis for >15 h undergo apoptosis shortly after mitotic exit, whereas those cells blocked in mitosis for <15 h showed variable fates with some cells living for days after mitotic exit [43]. This analysis was carried out in HeLa cells, as done in the present study. In contrast to these findings, the MiTMABs, which block cytokinesis, did not trap cells at this mitotic stage for a long period of time, but only slightly delayed mitotic exit by approximately 30 mins [4]. Nevertheless, time-lapse analysis indicated that every MiTMAB treated HeLa cell failing cytokinesis proceeded to apoptotic cell death approximately 7-10 hours after exiting mitosis. Conversely, we have previously shown that H460 cells spend a prolonged period of time trapped in cytokinesis in the presence of MiTMABs (up to 24 h) [4] and these cells remained viable during the following 24 h time period of analysis. Thus, in the case of the MiTMAB-based dynamin inhibitors, the induction of apoptosis appears to correlate with a short (rather than long) period of time that cells spend trapped in cytokinesis. The significance of this correlation needs to be investigated in more detail. Rather, the difference in apoptotic response between these two cell lines likely represents the underlying difference in their molecular components, such as p53 status and Bcl-2 protein levels.

Several reports suggest that p53 status is critical for determining the cellular response following polyploidization [21,44,45]. It is possible that MiTMAB-induced cell death is influenced by p53 status since its expression or mutation status also correlated with sensitivity (HeLa: p53^wt ^but almost undetectable levels due to HPV, HT29: p53^mut ^and SW480: p53^mut^) and resistance (MCF-7 and H460 contain p53^wt^) to apoptosis. Given that this gene is frequently lost or mutated in cancers [46], the ability of dynamin inhibitors to induce apoptosis following polyploidization in cells lacking functional p53 could be a favourable characteristic as a potential chemotherapeutic agent. It could be particularly relevant to those drug resistant cancers that often develop following p53 mutation. However, the contribution of p53 in determining the cellular response following polyploidization is under debate and is complicated by its multiple roles. For example, in response to aurora kinase inhibitors, p53^wt ^is required for G_1 _arrest of tetraploid cells [21] and for inducing apoptosis following tetraploid formation [45]. Therefore, p53 status alone is not the sole predictor of the cellular response following polyploidization.

The expression of Bcl-2 and Mcl-1, but not Bcl-XL, appears to correlate with the ability of cells to undergo apoptosis following exposure to MiTMABs. There are six anti-apoptotic Bcl-2 family members identified and several of these appear to contribute to drug resistance in cancer cells [37,38], suggesting that inhibition of multiple Bcl-2 family members will be necessary to achieve an optimal therapeutic effect. The development of antagonists toward Bcl-2 [47] and Mcl-1 [48] provide an attractive hypothesis that MiTMABs may synergise with these antagonists to sensitise resistant cell lines to undergo apoptosis. In line with this idea, the Bcl-2 antagonists, ABT-737 or ABT-263, have been shown to synergise with Plk and aurora kinase inhibitors [49] as well as conventional chemotherapeutic drugs, such as vincristine, *in vitro *and *in vivo *[50].

## Conclusions

Overall, our findings demonstrate that the MiTMAB family of dynamin inhibitors induce apoptosis in a concentration-dependent manner following polyploidization. More specifically, these are the first reported targeted anti-mitotic compounds which induce polyploidy by exclusively blocking cytokinesis. Thus, dynamin inhibitors are a new class of anti-mitotic compounds with potential anti-cancer action. MiTMAB-induced apoptosis is not only dependent on cytokinesis failure and polyploidization but also on specific molecular components of the apoptotic machinery, such as Bcl-2. Thus, inhibitors of these anti-apoptotic proteins, such as the Bcl-2 inhibitor ABT-737, may act synergistically with the MiTMAB dynamin inhibitors, broadening their therapeutic potential for the treatment of cancer.

## Authors' contributions

SJ designed and conducted experiments and carried out data analysis. AB and PJR participated in intellectual discussion of the data and manuscript writing. MC contributed to experimental design, co-ordination of the project, data analysis and manuscript writing. All authors read and approved the manuscript.

## Competing interests

The authors declare that they have no competing interests.
